# Supplementary material for: Enhancing alphafold-multimer-based protein complex structure prediction with MULTICOM in CASP15
Source: Commun Biol. 2023 Nov 10;6:1140. doi: 10.1038/s42003-023-05525-3 (PMC10638423; doi:10.1038/s42003-023-05525-3)
Supplement: Supplementary file 2 — Supplementary Information [file 42003_2023_5525_MOESM2_ESM.pdf]

Supplementary Materials

Table S1. The average TM-score of the best of five predictions of the top 15 out of 26 server predictors including NBIS-AF2-multimer (the standard AlphaFold-Multimer predictor) on the 41 multimers, 14 TBM multimers, 27 TBM/FM and FM multimers. When calculating the average TM-score, if a predictor did not submit a prediction for a target, the TM-score is set to 0. The bold font highlights the best result. The underline denotes the second best result.

Server predictor	Sum of Z-scores (> 0.0)	Avg TM-score on 41 multimers	Avg TM-score on 14 TBM multimers	Avg TM-score on 27 FM and FM/TBM multimers	Target count
Yang-Multimer	**28.4019**	0.7542	**0.8619**	0.6984	39
MULTICOM_deep	24.6154	0.7781	0.8536	0.7389	41
MULTICOM_qa	23.1823	0.7963	0.8541	0.7663	41
Manifold-E	22.8345	**0.8161**	0.8504	**0.7984**	41
DFolding-server	20.2187	0.6816	0.7835	0.6287	36
ColabFold	18.3899	0.6715	0.7584	0.6265	39
UltraFold_Server	18.2015	0.7641	0.7999	0.7456	41
MultiFOLD	17.4288	0.6900	0.7671	0.6500	41
Kiharalab_Server	16.561	0.6995	0.7839	0.6557	40
MUFold	16.4772	0.7478	0.8481	0.6958	41
NBIS-AF2-multimer	14.8905	0.7375	0.8438	0.6824	41
RaptorX-Multimer	14.7853	0.7061	0.8168	0.6487	40
DFolding-refine	12.8844	0.6973	0.8362	0.6253	36
GuijunLab-DeepDA	12.6262	0.7361	0.8309	0.6870	41
Yang-Server	12.2828	0.3839	0.6557	0.2430	19

Table S2. 19 kinds of combinations of MSA_{paired} and structural templates for homo-multimer targets.

MSA and Template Combinations	MSA_{paired}		Structural templates
	Sequence database	Interaction source	Template database
default_multimer	UniRef30, BFD, MGnify clusters	-	pdb70
default_pdb	UniRef30, BFD, MGnify clusters	-	pdb_sort90
default_pdb70	UniRef30, BFD, MGnify clusters	-	pdb70
default_comp	UniRef30, BFD, MGnify clusters	-	pdb_complex
default_struct	UniRef30, BFD, MGnify clusters	-	pdb_complex
default_af	UniRef30, BFD, MGnify clusters	-	Predicted chain structures

default_img	UniRef90, Integrated Microbial Genomes (IMG), metagenome sequence databases	-	pdb70
uniclust_oxmatch_a3m	UniClust30	Species annotation	pdb70
spec_iter_uniref_a3m	UniRef30	Species annotation	pdb70
spec_iter_uniref_sto	UniRef90	Species annotation	pdb70
spec_iter_uniprot_sto	UniProt	Species annotation	pdb70
spec_pdb	UniRef30	Species annotation	pdb_sort90
spec_pdb70	UniRef30	Species annotation	pdb70
spec_comp	UniRef30	Species annotation	pdb_complex
spec_struct	UniRef30	Species annotation	pdb_complex
spec_af	UniRef30	Species annotation	Predicted chain structures
pdb_iter_uniref_a3m	UniRef30	PDB	pdb70
pdb_iter_uniref_sto	UniRef90	PDB	pdb70
pdb_iter_uniprot_sto	UniProt	PDB	pdb70

Table S3. 29 kinds of combinations of MSA_{paired} and structural templates for hetero-multimer targets. The $MSA_{unpaired}$ for hetero-multimers is always generated by the same default MSA generation procedure in AlphaFold2-Multimer (e.g., searching the subunit/chain sequence against UniRef30 and BFD, and MGnify clusters to generate MSAs).

MSA & Template Combination	MSA_{paired}		Structural template	
	Sequence database	Interaction source	Template database	Concatenation
default_multimer	UniProt	Species annotation	pdb_seqres	-
default_pdb	UniProt	Species annotation	pdb_sort90	PDB code
default_pdb70	UniProt	Species annotation	pdb70	PDB code
default_comp	UniProt	Species annotation	pdb_complex	PDB code
default_struct	UniProt	Species annotation	pdb_complex	PDB code
default_af	UniProt	Species annotation	Predicted chain structures	-
uniclust_oxmatch_a3m	UniClust30	Species annotation	pdb_seqres	-
spec_iter_uniref_a3m	UniRef30	Species annotation	pdb_seqres	-
spec_iter_uniref_sto	UniRef90	Species annotation	pdb_seqres	-
spec_iter_uniprot_sto	UniProt	Species annotation	pdb_seqres	-
spec_pdb	UniRef30	Species annotation	pdb_sort90	PDB code
spec_pdb70	UniRef30	Species annotation	pdb70	PDB code
spec_comp	UniRef30	Species annotation	pdb_complex	PDB code
spec_struct	UniRef30	Species annotation	pdb_complex	PDB code

spec_af	UniRef30	Species annotation	Predicted chain structures	-
unidist_uniref_a3m	UniRef30	UniProt accession ID	pdb_seqres	-
unidist_uniref_sto	UniRef90	UniProt accession ID	pdb_seqres	-
unidist_uniprot_sto	UniProt	UniProt accession ID	pdb_seqres	-
str_iter_uniref_a3m	UniRef30	STRING database	pdb_seqres	-
str_iter_uniref_sto	UniRef90	STRING database	pdb_seqres	-
str_iter_uniprot_sto	UniProt	STRING database	pdb_seqres	-
str_pdb	UniRef90	STRING database	pdb_sort90	PDB code
str_pdb70	UniRef90	STRING database	pdb70	PDB code
str_comp	UniRef90	STRING database	pdb_complex	PDB code
str_struct	UniRef90	STRING database	pdb_complex	PDB code
str_af	UniRef90	STRING database	Predicted chain structures	PDB code
pdb_iter_uniref_a3m	UniRef30	PDB	pdb_seqres	-
pdb_iter_uniref_sto	UniRef90	PDB	pdb_seqres	-
pdb_iter_uniprot_sto	UniProt	PDB	pdb_seqres	-

Table S4. The four sources of protein-protein interaction information used with four sequence databases for generating 13 kinds of paired MSAs for multimers in total.

**Sources of interaction**	**Interaction identifier**	**Sequence database**	**Number of kinds of paired MSAs**
Species annotation	Organism identifier (OX), Organism name (OS), Taxonomy identifier (Tax)	UniClust30, UniRef30, UniRef90, UniProt	4
UniProt accession number	Distance between UniProt accession numbers	UniRef30, UniRef90, UniProt	3
STRING database	Interaction score	UniRef30, UniRef90, UniProt	3
PDB complex	Same PDB code	UniRef30, UniRef90, UniProt	3

Table S5. The differences of the MULTICOM server predictors (MULTICOM_deep and MULTICOM_qa) and MULTICOM human predictors (MULTICOM and MULTICOM_human).

	MULTICOM_deep	MULTICOM_qa	MULTICOM	MULTICOM_human
Number of predictions	15 - 195	15 - 195	40 - 315	40 - 315

Including refined predictions generated by FSAMR	No	No	Yes	Yes
Quality assessment method	AlphaFold-Multimer confidence score	Average of AlphaFold-Multimer confidence score and PSS score	PSS score	Average of AlphaFold-Multimer confidence score and PSS score

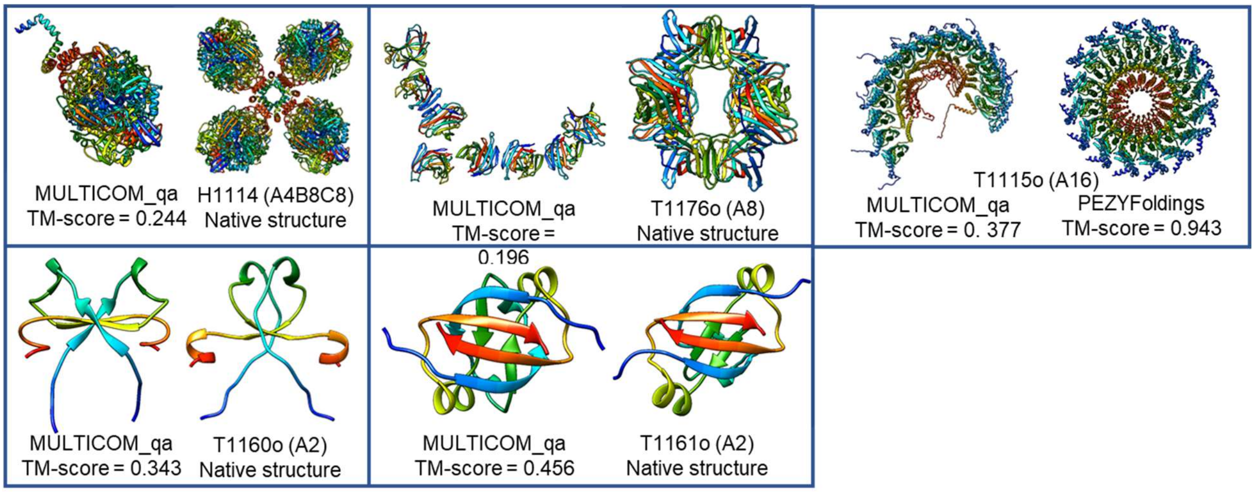

Figure S1. Structural comparison of MULTICOM_qa's best predictions to native structures for 5 CASP15 multimer targets. The structural comparison between the best structural prediction submitted from MULTICOM_qa and the native structure of H1114, T1176o, T1115o, T1160o and T1161o.

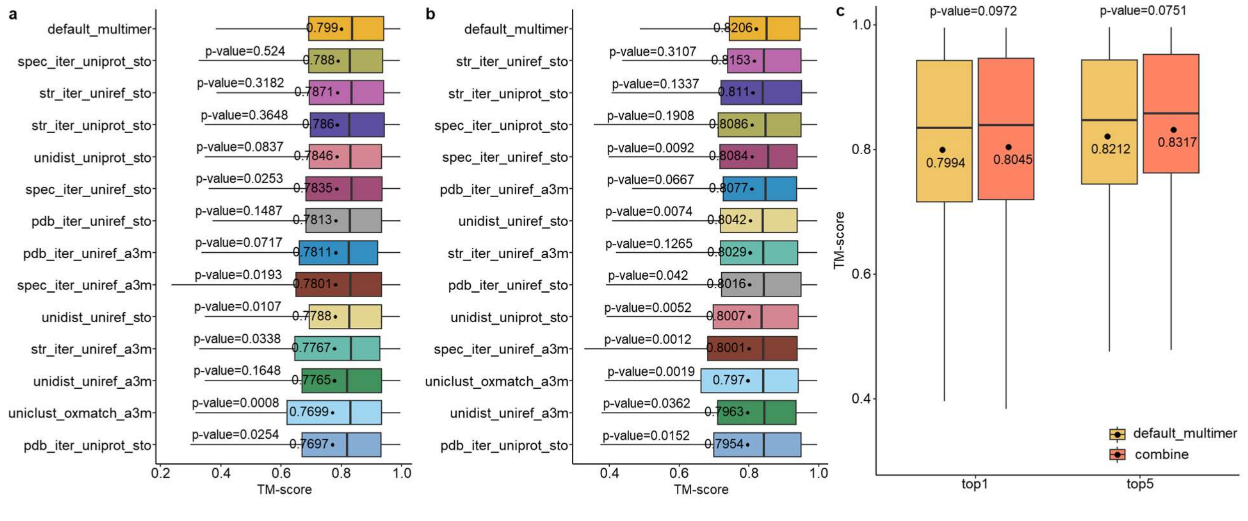

Figure S2. Comparative analysis of 14 kinds of MSA_{paired} on 100 hetero-multimers based on TM-scores. (**a**) The average TM-score of top-1 predictions generated from 14 kinds of MSA_{paired} and selected by AlphaFold-Multimer's confidence score on the 100 hetero-multimers. The per-target mean (or median) TM-score of each kind of MSA_{paired} is located by the black dot in the box (or by a vertical line). The p-value of the one-sided Wilcoxon signed rank test between default_multimer and each kind of MSA_{paired} is reported.

(**b**) The average TM-score of best of top-5 predictions generated from the 14 kinds of MSA_{paired} on the 100 hetero-multimers. The per-target mean (or median) TM-score of each kind of MSA_{paired} is located by the black dot in the box (or by a vertical line). The p-value of the one-sided Wilcoxon signed rank test between default_multimer and each kind of MSA_{paired} is also reported. (**c**) The comparison between the average TM-score of top-1 (best of top 5) predictions from the combined model pool of the 13 kinds of MSA_{paired} (denote as *combine*) and that of default_multimer. The per-target mean (or median) TM-score of each kind of MSA_{paired} is located by the black dot in the box (or by a horizontal line). The p-value of the one-sided Wilcoxon signed rank test between combine and default_multimer is reported on top of the box.

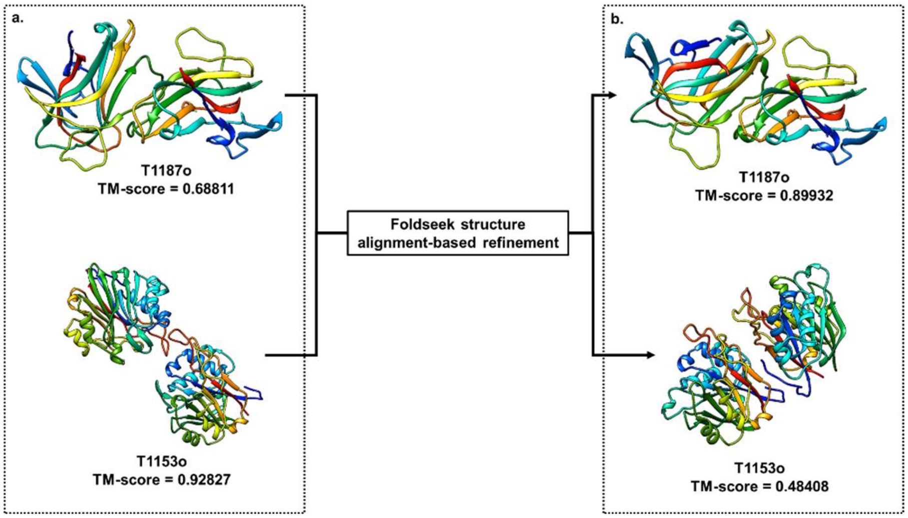

Figure S3. A good example (T1187o) and a bad example (T1153o) for the Foldseek structure alignment-based multimer structure refinement (FSAMR). **a**: the prediction before the refinement; **b**: the prediction after the refinement.

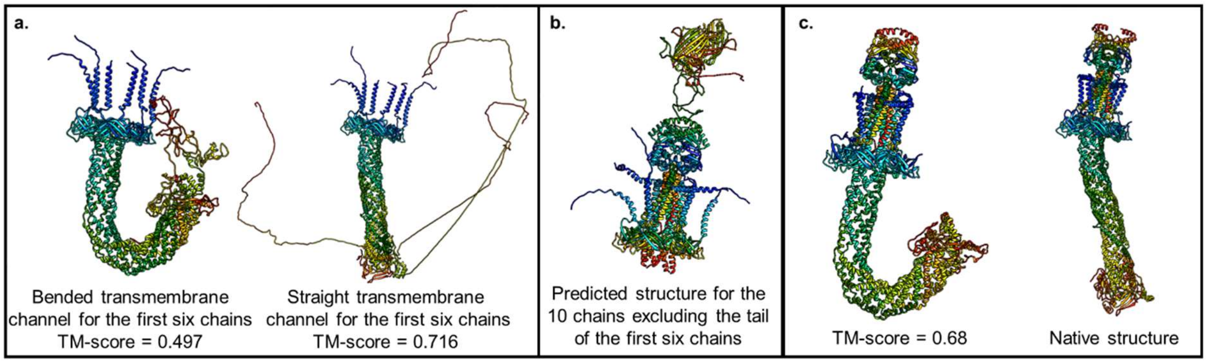

Figure S4. Complex structure predictions and comparison for H1137 chains. **a.** The two conformations (with TM-score = 0.497 and 0.716) of predicted complex structure for the first six chains of H1137 (A1B1C1D1E1F1); **b.** The predicted structure for the 10 chains excluding the tail of the first six chains; **c.** The native structure and the full-length complex structure (TM-score = 0.68) for H1137 built from the bended transmembrane channel for the first six chains (**structure in plot a**) and the predicted structure for the 10 chains excluding the tail of the first six chains (**structure in plot b**).

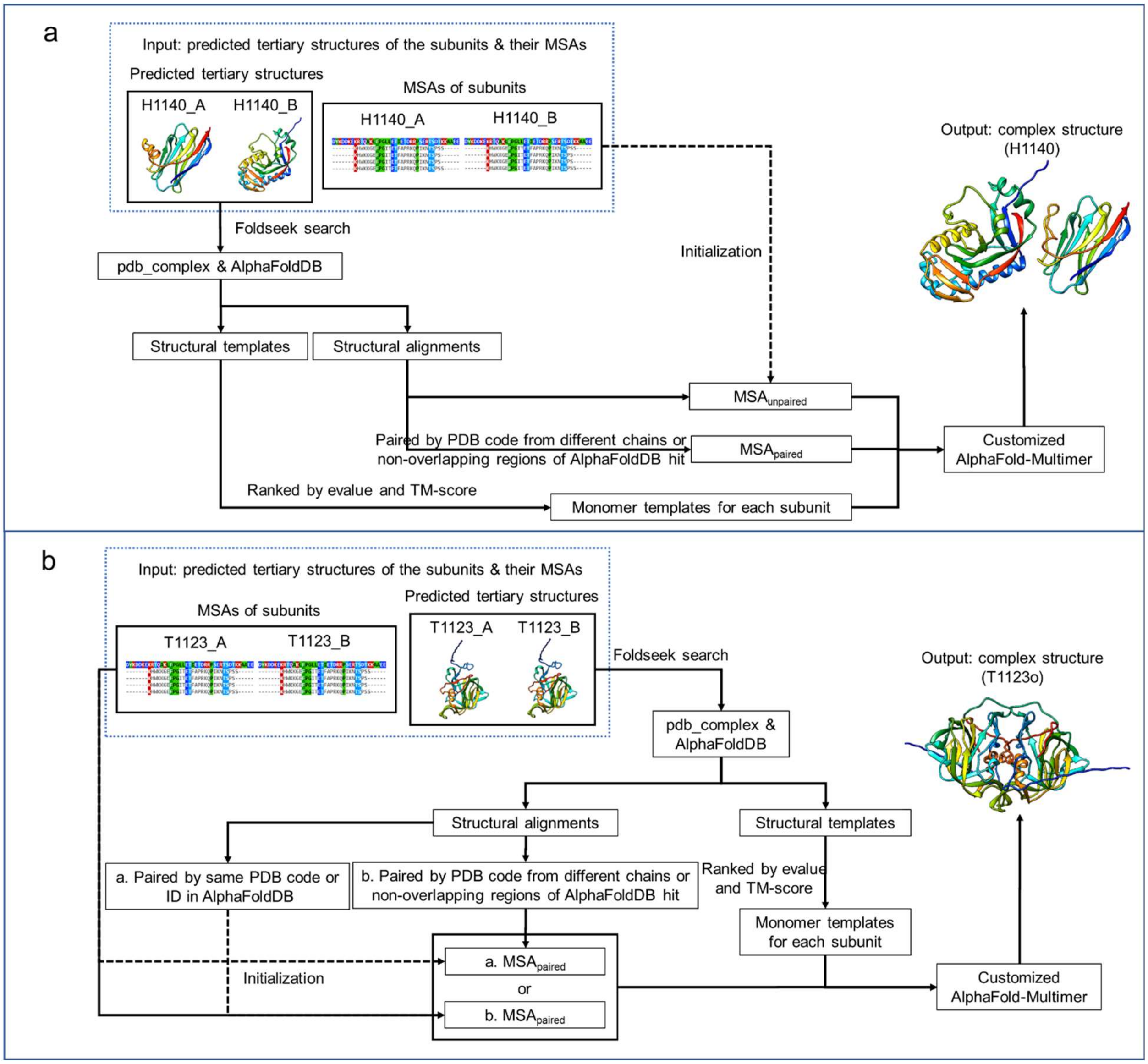

Figure S5. The illustration of the workflow of Foldseek structure alignment-based multimer structure generation (FSAMG). **(a)** a CASP15 hetero-dimer target H1140 and **(b)** a CASP15 homo-dimer target T1123o as examples. The black dash line denotes for the initialization to the $MSA_{unpaired}$ for hetero-multimer and MSA_{paired} for homo-multimer.
