# Supplementary material for: Enhancing alphafold-multimer-based protein complex structure prediction with MULTICOM in CASP15
Source: Commun Biol. 2023 Nov 10;6:1140. doi: 10.1038/s42003-023-05525-3 (PMC10638423; doi:10.1038/s42003-023-05525-3)
Supplement: Supplementary file 3 — Description of Additional Supplementary Files [file 42003_2023_5525_MOESM3_ESM.pdf]

Description of Additional Supplementary Files

File name: Supplementary Data 1

Description: The data used to draw the figures and tables in the main manuscript.
